# Evaluating the Effects of Tetrachloro-1,4-benzoquinone, an Active Metabolite of Pentachlorophenol, on the Growth of Human Breast Cancer Cells

**DOI:** 10.1155/2016/8253726

**Published:** 2016-02-14

**Authors:** Binbing Ling, Bosong Gao, Jian Yang

**Affiliations:** Drug Discovery and Development Research Group, College of Pharmacy and Nutrition, University of Saskatchewan, 107 Wiggins Road, Saskatoon, SK, Canada S7N 5E5

## Abstract

Tetrachloro-1,4-benzoquinone (TCBQ), an active metabolite of pentachlorophenol (PCP), is genotoxic and potentially carcinogenic. As an electrophilic and oxidative molecule, TCBQ can conjugate with deoxyguanosine in DNA molecules and/or impose oxidative stress in cells. In the current study, we investigated the effects of TCBQ on intracellular ROS production, apoptosis, and cytotoxicity against three different subtypes of human breast cancer cells. Luminal A subtype MCF7 (ER^+^, PR^+^, HER2^−^) cells maintained the highest intracellular ROS level and were subjected to TCBQ-induced ROS reduction, apoptosis, and cytotoxicity. HER2 subtype Sk-Br-3 (ER^−^, PR^−^, HER2^+^) cells possessed the lowest intracellular ROS level. TCBQ promoted ROS production, inhibited apoptosis, and elevated cytotoxicity (due to necrosis) against Sk-Br-3 cells. Triple-negative/basal-like subtype MDA-MB-231 cells were less sensitive towards TCBQ treatment. Therefore, the effect of prolonged exposure to PCP and its active metabolites on cancer growth is highly cancer-cell-type specific.

## 1. Introduction

Pentachlorophenol (PCP), a potent uncoupler of oxidative phosphorylation, was widely used as a low-cost and effective farm pesticide in agriculture and wood preservative in timber industry in the last century [1–5]. Because of its high toxicity to fish, farm animals, and human, PCP was banned from agricultural usage in the 1980s [3, 5–8]. PCP is highly resistant to biodegradation due to the introduction of high and obstructive halogenation, making it one of the most persistent pollutants in the environment [9, 10]. Furthermore, PCP is reasonably soluble (10–20 mg/L) and can be spread to unpolluted areas* via* rain or human activities, making it a continuous source of contamination to fruits, vegetables, and grains [3, 11, 12].

The daily net intake of PCP is about 0.05 *μ*g/kg and 16 *μ*g/kg of body weight for Canadians and Americans, respectively [12, 13]. However, it could reach as high as 24,000 *μ*g for people occupationally exposed to PCP (i.e., 282 *μ*g/kg for a man with the Canadian national average body weight of 85 kg and 343 *μ*g/kg for a woman with the Canadian national average body weight of 70 kg) [14]. Because of its high lipophilicity, PCP can easily cross skin, respiratory tract, and gastrointestinal tract and be distributed in different tissues [3]. The half-life (*t*_1/2_) of PCP ranges from 33 hours to 16 days in human bodies [14]. Liver and kidney contain the highest levels after PCP exposures [14]. Extended exposure to PCP may cause serious diseases such as neurological disorders, immune disorders, and cancers [15–17]. PCP was also found in breast milk and could be passed to infants by breastfeeding [18, 19]. PCP exposure has been implied as a causal factor for women's repeated miscarriages, endocrine disorders, and even breast cancer [20–22]. The toxicity of PCP is most likely due to the formation of a highly reactive metabolite, tetrachloro-1,4-benzoquinone (TCBQ) [23, 24]. As an electrophilic molecule, TCBQ forms adducts with deoxyguanosine in DNA molecules, causing genotoxic effects to cells [23–25]. Furthermore, TCBQ is susceptible to quick reduction to generate tetrachlorosemiquinone (TCSQ) radicals and imposes oxidative stress in cells. It has been shown that TCBQ increased the intracellular ROS level by about 10-fold in human hepatoma HepG2 cells after 24 h of exposure [23, 24]. These studies implicate that TCBQ is genotoxic and potentially carcinogenic to both human and animals.

In contrast to the vast studies on genotoxic and cytotoxic effects of PCP and its reactive metabolite TCBQ to normal human cells, little is known on how continued exposure to PCP or TCBQ could affect the growth of cancer cells. As a genotoxic and oxidative compound, TCBQ may be a double-edged sword. On the one hand, it may initiate carcinogenesis in normal cells and/or promote cancer cell growth by elevating the intracellular ROS level. On the other hand, TCBQ may cause cell death* via* forming adducts with cancer cell DNA molecules and/or induce cell apoptosis through increasing the intracellular ROS level above the apoptotic threshold in cancer cells. To gain an insight into how extended PCP exposure could affect tumor growth for women with breast cancer, we undertook an* in vitro* study to elucidate the effects of TCBQ on oxidative stress, apoptosis, and cytotoxicity against human breast cancer cells. In spite of big differences in morphology, growth, survival, migration, invasiveness, and metastasis, breast cancer cells are commonly divided into 4 subtypes, luminal A (ER^+^ and/or PR^+^, HER2^−^), luminal B (ER^+^ and/or PR^+^, HER2^+^), HER2 (ER^−^, PR^−^, HER2^+^), and triple-negative/basal-like (ER^−^, PR^−^, HER2^−^), based on expression of three cell surface receptors, estrogen receptor (ER), progesterone receptor (PR), and HER2/neu receptor (HER2). The weakly invasive luminal A subtype MCF7 (ER^+^, PR^+^, HER2^−^) cell line, weakly invasive HER2 subtype Sk-Br-3 (ER^−^, PR^−^, HER2^+^), and highly invasive triple-negative MDA-MB-231 (ER^−^, PR^−^, HER2^−^) were selected for this study.

## 2. Materials and Methods

### 2.1. Materials

TCBQ and 2′,7′-dichlorofluorescein diacetate (DCFH-DA) were purchased from Sigma-Aldrich Canada (Oakville, ON, Canada). Human breast cancer cell lines MCF7 (ER^+^, PR^+^, HER2^−^), Sk-Br-3 (ER^−^, PR^−^, HER2^+^), and MDA-MB-231 (ER^−^, PR^−^, HER2^−^) were purchased from the American Type Culture Collection (ATCC) (Manassas, VA, USA). ATCC-recommended cell culture media for each cell line were purchased from Cedarlane Canada (Burlington, ON, Canada). Cell apoptosis assay kit, Caspase-Glo® 3/7 Assay, and cytotoxicity assay kit, CytoTox96® Nonradioactive Cytotoxicity Assay, were purchased from Promega Corporation (Madison, WI, USA).

### 2.2. Cell Culture

Human breast cancer cell lines MCF7, Sk-Br-3, and MDA-MB-231 were cultured in T-75 culture flasks under ATCC-recommended cell culture conditions at 37°C in a Forma™ Series II Water-Jacketed CO_2_ Incubator from ThermoFisher Scientific Inc. (Waltham, MA, USA). Cell lines MCF7 and Sk-Br-3 were cultured with 5% CO_2_, whereas cell line MDA-MB-231 was cultured with 0% CO_2_. Culture media were changed every 2-3 days for each cell line.

### 2.3. Intracellular ROS Measurement

All experiments in the current study were carried out in triplicate. Intracellular ROS level was measured using probe DCFH-DA in the MCF7, Sk-Br-3, and MDA-MB-231 cells with and without TCBQ treatment under normoxic condition. DCFH-DA was prepared in stock solution of 10 mM in dimethyl sulfoxide (DMSO). Working solution of DCFH-DA was prepared by diluting the stock solution with the respective cell culture media with a final concentration of 0.1 mM. Cells of each cell line were plated on a black flat-bottom 96-well plate at 10,000 cells per well and incubated at 37°C for 18 h. Working solution (5 *μ*L) was added to each well and allowed to react with the cells for 30 min before being aspirated out. The cells were then washed with 200 *µ*L 1x PBS (phosphate buffered saline) buffer twice. Finally, 100 *µ*L 1x PBS buffer was added to each well and fluorescence was read at extinction of 485 nm and emission of 528 nm using an Agilent 8453E UV-visible Spectroscopy System (Agilent Technologies Canada, Mississauga, ON, Canada).

### 2.4. Apoptosis and Cytotoxicity Assays

The cultured breast cancer MCF7, Sk-Br-3, or MDA-MB-231 cells were plated in 96-well plates (10,000 cells/well) and grown to 70–80% confluence before being treated with TCBQ (final concentrations: 0.16 *µ*M, 0.31 *µ*M, 0.63 *µ*M, 1.25 *µ*M, 2.5 *µ*M, 5 *µ*M, and 10 *µ*M) for 18 h. The optimal exposure time was determined by a pilot study. Treatment with DMSO, in which TCBQ stock solution was prepared, was used as negative control. Apoptosis (caspase 3/7 level) and cytotoxicity (lactate dehydrogenase level) were measured using the Caspase-Glo 3/7 Assay and the CytoTox96 Nonradioactive Cytotoxicity Assay, respectively.

### 2.5. Statistical Analyses

The experimental data were processed using Microsoft Excel 2010 and presented as mean ± standard deviation. One-way ANOVA with Dunnett's comparison as posthoc analysis was performed with GraphPad Prism 6 (GraphPad Software, Inc., La Jolla, CA, USA). A* P* value of less than 0.05 was considered to be statistically significant.

## 3. Results and Discussion

### 3.1. Intracellular ROS Level under Normoxia

**Figure 1:**
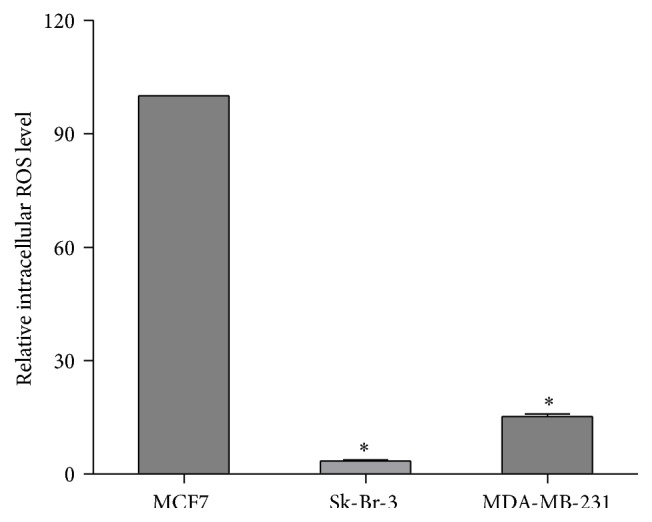
Relative intracellular ROS level in human breast cancer MCF7, Sk-Br-3, and MDA-MB-231 cells under normoxic condition. The highest intracellular ROS level was observed in MCF7 cells and set as 100.

Reactive oxygen species (ROS), which are short-lived and normally generated as byproducts of mitochondrial energy metabolism, play important roles in cell growth, cell signaling, and homeostasis in normal cells [26–28]. Persistently elevated ROS level is a characteristic phenomenon for tumorigenesis, tumor growth, and cancer metastasis [26, 29]. However, measuring and comparing the intracellular ROS across different types of cancer cells or tissues is a challenging task as the ROS level is significantly influenced by the cancer microenvironment, intracellular signaling regulation, and the type and degree of hypoxia. In the current study, we measured the intracellular ROS level in human breast cancer MCF7, Sk-Br-3, and MDA-MB-231 cells under normoxic condition. Although our measurement may not necessarily represent the pathophysiological situation, it allowed us to cross-compare the intracellular ROS of different types of breast cancer cells on the same scale and make reasonable prediction on the effects of exogenously administered agents such as oxidative compounds and chemotherapy drugs on ROS production. As shown in Figure 1, the weakly invasive luminal A subtype MCF7 cells maintained the highest intracellular ROS level among all three cancer cell lines. The respective intracellular ROS level in the weakly invasive HER2 subtype Sk-Br-3 cells and highly invasive triple-negative MDA-MB-231 cells was only about 3.5 ± 0.3% (*P* < 0.01) and 15.3 ± 0.6% (*P* < 0.01) of that in the MCF7 cells. Our study results were consistent with observation that Sk-Br-3 and MDA-MB-231 cells exhibited a much lower basal oxygen consumption level, relied more on glycolysis rather than oxidative phosphorylation for energy production, and had much higher uptake of F^18^-fluorodeoxyglucose (FDG) than MCF7 cells [30–32]. Lower oxidative phosphorylation would lead to less ROS in Sk-Br-3 and MDA-MB-231 cells. Furthermore, MCF7 cells are likely more tolerable to ROS and may have a much higher apoptotic threshold than Sk-Br-3 and MDA-MB-231 cells.

### 3.2. Effects of TCBQ on the Intracellular ROS Level

**Figure 2:**
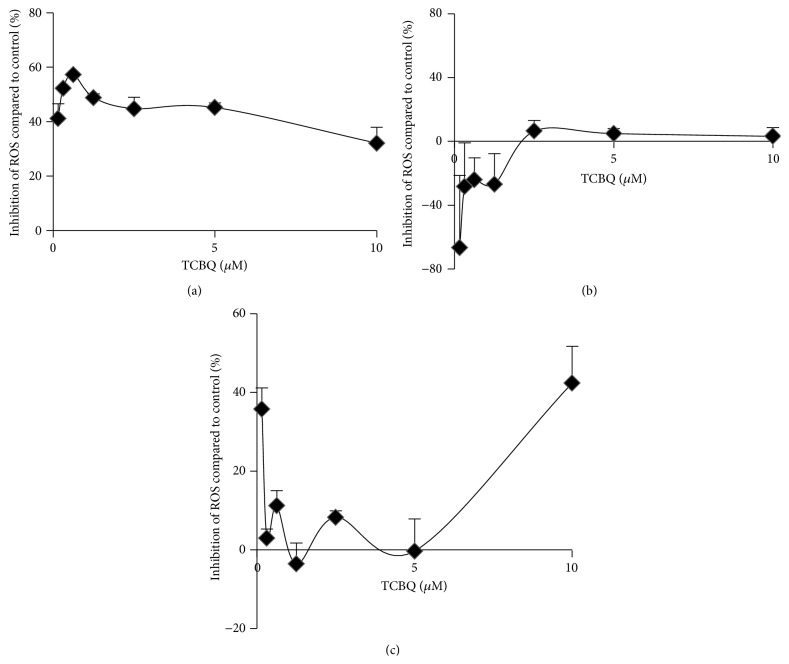
Effects of TCBQ on intracellular ROS production in human breast cancer MCF7 (a), Sk-Br-3 (b), and MDA-MB-231 (c) cells under normoxic condition. The concentration of TCBQ was 0.16 *µ*M, 0.31 *µ*M, 0.63 *µ*M, 1.25 *µ*M, 2.5 *µ*M, 5 *µ*M, and 10 *µ*M, respectively. Treatment with DMSO was used as negative control. The inhibition of ROS production (%) compared to control was statistically significant at all TCBQ concentrations towards the MCF7 cells, statistically insignificant at all TCBQ concentrations towards the Sk-Br-3 cells, and statistically significant at TCBQ concentration of 0.16 *µ*M and 10 *µ*M towards the MDA-MB-231 cells. Statistical analysis was performed by one-way ANOVA with Dunnett's comparison as posthoc analysis.

As an oxidative compound, TCBQ was shown to increase the intracellular ROS level by almost 10-fold in human hepatoma HepG2 cells at 5 *µ*M concentration after 24 h of exposure [23]. Higher concentration of TCBQ did not further enhance ROS generation, implicating that TCBQ has already reached a plateau for its function on ROS production in HepG2 cells. Therefore, we examined the effect of TCBQ on ROS production in MCF7, SK-Br-3, and MDA-MB-231 cells with its concentration ranging from 0.16 *µ*M to 10 *µ*M (~39 *μ*g/kg to 2459 *μ*g/kg, covering the range of previously reported PCP exposure levels and assuming all PCP could be metabolized to TCBQ quickly). Interestingly, TCBQ inhibited instead of promoting ROS production in MCF7 and MDA-MB-231 cells (Figures 2(a) and 2(c)). MCF7 cells gave a bell-shaped response towards TCBQ with the maximum inhibition of ROS production (57% decrease compared to the control, statistically significant) at 0.63 *µ*M, whereas MDA-MB-231 showed a U-shaped response towards TCBQ treatment with approximately 36% and 42% reduction of ROS compared to the control (statistically significant) at 0.16 *µ*M and 10 *µ*M, respectively. As for Sk-Br-3 cells, TCBQ increased the intracellular ROS production in a concentration-dependent manner with ROS production dwindled along with elevated TCBQ concentration (Figure 2(b)). The intracellular ROS level was increased by 66% compared to the control at 0.16 *µ*M of TCBQ; and TCBQ lost its function on ROS production when its concentration surpassed 2 *µ*M. However, the change in ROS level compared to control was statistically insignificant at all TCBQ concentrations. Recently, it was reported that quinone compounds were able to regulate ROS production both positively and negatively in human HEK293 cells [33]. Our current results reinforced and complemented to this study that the effect of quinones on ROS production is highly compound-type specific and cell-type specific. However, it is unknown how TCBQ decreased ROS production in the MCF7 and MDA-MB-231 cells and increased ROS production in the Sk-Br-3 cells even though the ROS increase was statistically insignificant. It has been reported in previous studies that the expression level of glutathione peroxidase (GPx) was much higher in Sk-Br-3 and MDA-MB-231 cells than MCF7 cells and the expression of GPx-1 was increased upon PCP treatment in murine melanoma B16F10 cells [34, 35]. Therefore, we speculated that the different effects of TCBQ on ROS production might be related to its capability of altering intracellular glutathione (GSH) level, which, in turn, is determined by the expression level of GSH-related enzymes such as GPx, glutathione reductase (GR), and glutathione S-transferase (GST). We will undertake further studies to confirm whether the TCBQ effect on ROS production is indeed* via* changing the expression of GSH-related enzymes.

### 3.3. Apoptotic Effects

**Figure 3:**
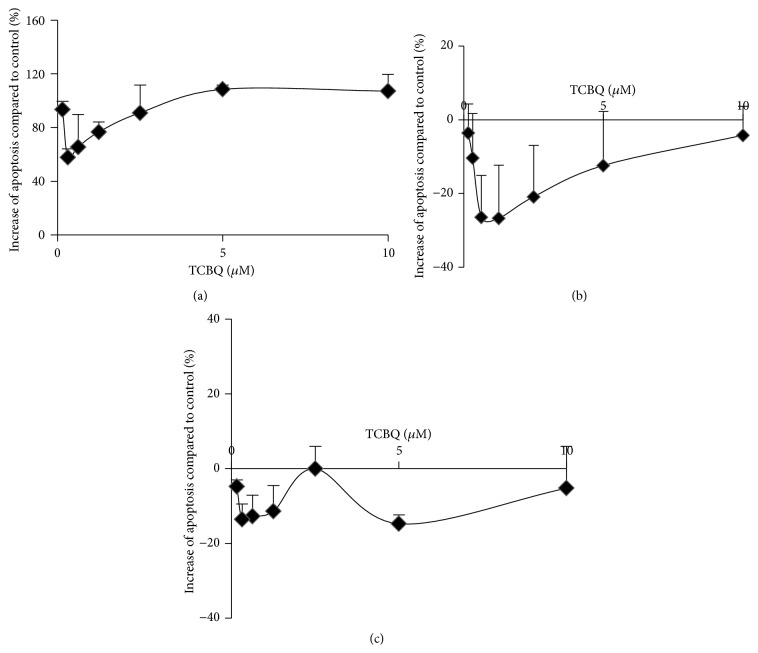
Apoptotic (caspase 3/7 level) effect of TCBQ towards human breast cancer MCF7 (a), Sk-Br-3 (b), and MDA-MB-231 (c) cells. The concentration of TCBQ was 0.16 *µ*M, 0.31 *µ*M, 0.63 *µ*M, 1.25 *µ*M, 2.5 *µ*M, 5 *µ*M, and 10 *µ*M, respectively. Treatment with DMSO was used as negative control. Apoptosis was increased upon TCBQ treatment towards the MCF7 cells. The increase of apoptosis (%) compared to control was statistically significant at all TCBQ concentrations. However, apoptosis was decreased upon TCBQ treatment against the Sk-Br-3 and MDA-MB-231 cells. The decrease of apoptosis (%) compared to control was statistically insignificant at all TCBQ concentrations towards both cell lines. Statistical analysis was performed by one-way ANOVA with Dunnett's comparison as posthoc analysis.

A very recent study showed that 1,4-benzoquinone (BQ) induced cell apoptosis in a concentration-dependent manner in mouse bone marrow cells [36]. However, another study reported that TCBQ induced oxidative stress but not apoptosis in male Kunming mice [37]. Low level of oxidative stress could trigger protein kinase D1- (PKD1-) mediated cell survival while high level of oxidative stress could initiate apoptosis* via* activating c-Jun N-terminal kinases (JNKs) to downregulate various antiapoptotic proteins [38–41]. Taking into consideration our current observed effects of TCBQ on ROS production, it is rational to hypothesize that the apoptotic effect of TCBQ is also likely to be cell-type specific. To examine our hypothesis, we measured the apoptotic effect (caspase 3/7 level) of TCBQ against the three breast cancer cell lines. As illustrated in Figure 3, cell apoptosis was increased in MCF7 cells (statistically significant) but decreased in Sk-Br-3 and MDA-MB-231 cells (statistically insignificant) throughout the TCBQ concentration range.

In general, MCF7 cells gave a concentration-dependent response towards TCBQ (Figure 3(a)). Apoptosis was increased by more than 59% compared to the control at TCBQ concentration of 0.31 *µ*M and reached a plateau of 108% as TCBQ concentration reached 5 *µ*M. We speculated that decreased ROS production in the MCF7 cells might alleviate PKD1 activation, which, in turn, elevated cell apoptosis* via* downregulating the expression of antiapoptotic proteins. As for Sk-Br-3 and MDA-MB-231 cells, apoptosis was reduced upon TCBQ treatment. Sk-Br-3 cells showed a reverse bell-shaped response towards TCBQ treatment with the maximum inhibition of apoptosis (~30%) at concentrations around 1 *µ*M (Figure 3(b)). It is possible that increase in intracellular ROS level upon TCBQ treatment triggered PKD1-mediated cell survival. MDA-MB-231 cells were insensitive to TCBQ treatment and maintained relatively flat inhibition of apoptosis of less than 15% (Figure 3(c)). It is highly doubtful that TCBQ adopted the same mechanism to elicit its antiapoptotic functions in Sk-Br-3 and MDA-MB-231 cells as it caused opposite effects on intracellular ROS production in these two types of cancer cells even though the antiapoptotic functions were not statistically significant. Further studies are required to confirm whether TCBQ possesses any antiapoptotic effects against the Sk-Br-3 and MDA-MB-231 cells under different culture conditions such as hypoxia and identify the underlying mechanism on how TCBQ prompts its proapoptotic or antiapoptotic functions towards different human breast cancer cells.

### 3.4. Cytotoxic Effects

**Figure 4:**
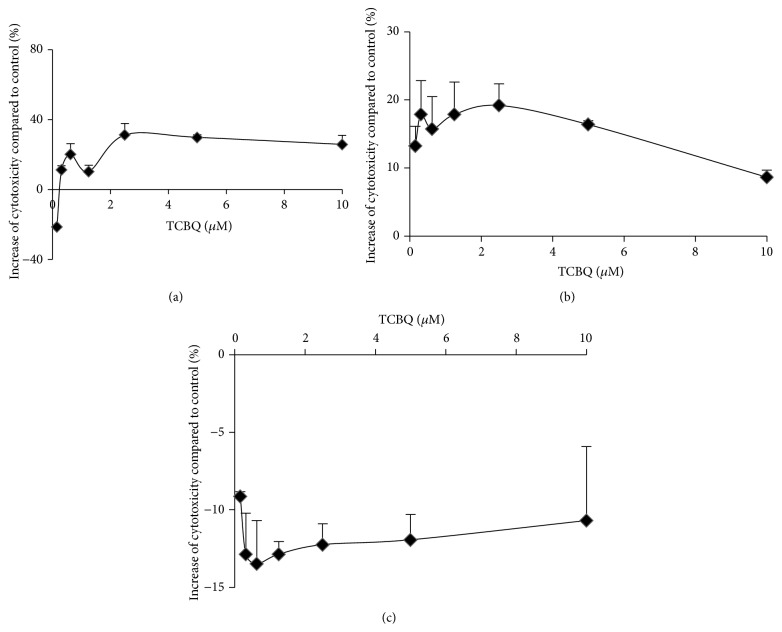
Cytotoxic effect of TCBQ towards human breast cancer MCF7 (a), Sk-Br-3 (b), and MDA-MB-231 (c) cells. The concentration of TCBQ was 0.16 *µ*M, 0.31 *µ*M, 0.63 *µ*M, 1.25 *µ*M, 2.5 *µ*M, 5 *µ*M, and 10 *µ*M, respectively. Treatment with DMSO was used as negative control. Cytotoxicity was increased upon TCBQ treatment against both MCF7 and Sk-Br-3 cells. The increase of cytotoxicity (%) compared to control was statistically significant at TCBQ concentration of 0.16 *µ*M, 0.63 *µ*M, 1.25 *µ*M, 2.5 *µ*M, and 5 *µ*M towards the MCF7 cells and at TCBQ concentration of 0.16 *µ*M, 0.31 *µ*M, 0.63 *µ*M, 1.25 *µ*M, 2.5 *µ*M, and 5 *µ*M towards the Sk-Br-3 cells, respectively. However, cytotoxicity was decreased upon TCBQ treatment towards the MDA-MB-231 cells. The decrease of cytotoxicity (%) compared to control was statistically significant at TCBQ concentration of 0.31 *µ*M, 0.63 *µ*M, 1.25 *µ*M, 2.5 *µ*M, 5 *µ*M, and 10 *µ*M. Statistical analysis was performed by one-way ANOVA with Dunnett's comparison as posthoc analysis.

As an active electrophilic molecule, TCBQ conjugates with 2′-deoxyguanosine of DNA molecules to form dichlorobenzoquinone-1,N^2^-etheno-2′-deoxyguanosine [25]. This conjugation reaction could initiate two opposite responses inside human body. Firstly, it may cause gene mutations, which could subsequently lead to carcinogenesis. However, it is still debatable whether chlorinated pesticides as well as their metabolites such as TCBQ could initiate carcinogenesis, as PCP was shown to promote rather than induce hepatocarcinogenesis in B6C3F_1_ mice [42]. Secondly, the conjugation between TCBQ and 2′-deoxyguanosine may provoke apoptosis and/or necrosis, a common mechanism adopted by alkylating chemotherapy drugs like carboplatin to kill cancer cells. Herein, we examined whether TCBQ could impose cytotoxic effect (necrosis + apoptosis) against the breast cancer cells. As shown in Figure 4, TCBQ was cytotoxic only to MCF7 (TCBQ concentration > 0.3 *µ*M) and Sk-Br-3 cells. MCF7 cells gave a log-shaped response towards TCBQ treatment with a maximum of 31% increase in cytotoxicity compared to the control at concentration higher than 2.5 *µ*M (Figure 4(a)). However, TCBQ exhibited cell-protective effect when its concentration was reduced to 0.16 *µ*M. It was unknown what factors contributed to the cell-protective (proliferation or survival) effects. The observed lower cytotoxic effects (less than 31% increase compared to control) than apoptotic effects (59–109% increase compared to control) were due to the different mechanism of the assay kits. The apoptosis assay kit, Caspase-Glo 3/7 Assay, measures the caspase 3/7 activity, whereas the cytotoxicity assay kit, CytoTox96 Nonradioactive Cytotoxicity Assay, measures lactate dehydrogenase (LDH) release upon cell lysis. Thus, at the end of 18 h of TCBQ treatment, some of the MCF7 cells that underwent apoptosis might be still alive with intact cell membranes, resulting in lower observed cytotoxicity. Sk-Br-3 cells showed a very shallow bell-shaped response towards TCBQ with a maximum of 19% increase in cytotoxicity at concentration of 2.5 *µ*M (Figure 4(b)). MDA-MB-231 cells were less sensitive to TCBQ treatment and maintained a stable marginal cell-protective effect (~12% decrease in cytotoxicity compared to the control, statistically significant except at 0.16 *µ*M) throughout the whole TCBQ concentration range (Figure 4(c)). Thus, the cytotoxic effect of TCBQ was as well cell-type specific against human breast cancer cells.

## 4. Conclusion

In the current study, we showed that effects of TCBQ, an active metabolite of PCP, on intracellular ROS production, apoptosis, and cytotoxicity were cell-type specific against human breast cancer. Triple-negative MDA-MB-231 cells were less sensitive to TCBQ treatment. Our results implicated that cell type was the decisive factor in determining whether continued exposure to PCP as well as its active metabolites such as TCBQ would promote or inhibit tumor growth. Of course, the exposure level of PCP also plays an important role in tumor growth. To our knowledge, this was the first study to examine how continued exposure of PCP could affect breast cancer cell growth* via* its highly reactive metabolite TCBQ and demonstrated that breast cancer cell type was a decisive factor for the PCP or TCBQ effects. Further cell line and mouse xenograft studies are warranted to establish a relationship between effect of continued PCP and TCBQ exposure and the four common breast cancer molecular subtypes (luminal A, luminal B, HER2, and triple-negative/basal-like). The underlying mechanisms for the effects of PCP and TCBQ on proliferation, apoptosis, and cytotoxicity, as well as metabolic profile of PCP in normal and breast cancer cells, will also be investigated.

## Abbreviations

ROS: Reactive oxygen species
ER: Estrogen receptor
PR: Progesterone receptor
HER2: HER2/neu receptor.
